# Concomitant Recurrent Pneumothorax and Takotsubo Cardiomyopathy in a Chronic Obstructive Pulmonary Disease Patient

**DOI:** 10.7759/cureus.30850

**Published:** 2022-10-29

**Authors:** Takayuki Takimoto, Takehiko Kobayashi, Shojiro Minomo

**Affiliations:** 1 Department of Internal Medicine, National Hospital Organization Kinki-Chuo Chest Medical Center, Sakai, JPN

**Keywords:** electrocardiogram, broken-heart syndrome, acute coronary syndrome, chronic obstructive pulmonary disease, takotsubo cardiomyopathy, pneumothorax

## Abstract

Chest pain is one of the major causes of emergency room visits. Here, we present the case of a patient with chest pain who developed recurrent pneumothorax and Takotsubo cardiomyopathy (TC). An 80-year-old man, receiving supplemental oxygen for chronic obstructive pulmonary disease (COPD), presented to the emergency room with chest pain and dyspnea. On examination, his chest pain was initially assessed to be secondary to recurrent pneumothorax. However, on further evaluation, an electrocardiogram (ECG) showed ST-segment elevation along with elevated troponin levels. Ultimately, he was diagnosed with TC. ECG, if indicated by echocardiography, should be considered to detect concomitant heart disease when dealing with pneumothorax. TC should be recognized as a cardiac disease that can be caused by pneumothorax.

## Introduction

Chest pain is one of the major causes of emergency room visits [1]. Although it can sometimes be a symptom of cardiac disease, many other possible causes include pulmonary embolism, pleuritis, and pneumothorax. Takotsubo cardiomyopathy (TC) is a unique type of cardiomyopathy characterized by left ventricular systolic dysfunction in association with stressful conditions [2-5]. Its presentation can mimic acute coronary syndrome (ACS) which usually requires immediate intervention. Here, we present the case of a patient with chest pain who developed recurrent pneumothorax and concomitant TC.

## Case presentation

**Figure 1:**
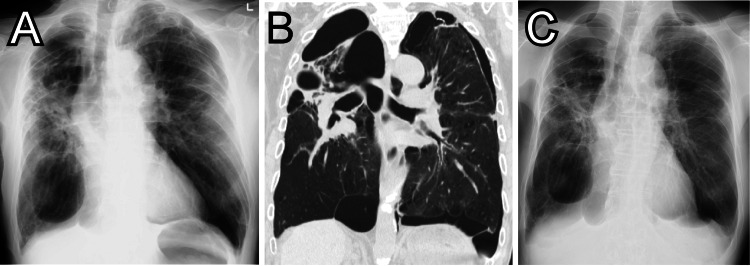
Chest X-ray and computed tomography. X-ray (A) and computed tomography (B) of the chest on arrival showing a left-sided mild pneumothorax and pre-existent multiple giant bullae. (C)  Chest X-ray after 10 days showing improvement of the left-sided pneumothorax.

An 80-year-old man, receiving supplemental oxygen (1 L/minute at rest) for chronic obstructive pulmonary disease (COPD), presented to the emergency room with chest pain and dyspnea. He had a history of repeated pneumothorax and previous myocardial infarction. On arrival, chest X-ray and computed tomography (CT) revealed pre-existent multiple giant bullae and a left-sided pneumothorax (Figure 1). Hence, we considered that the symptom was due to recurrent pneumothorax.

**Figure 2:**
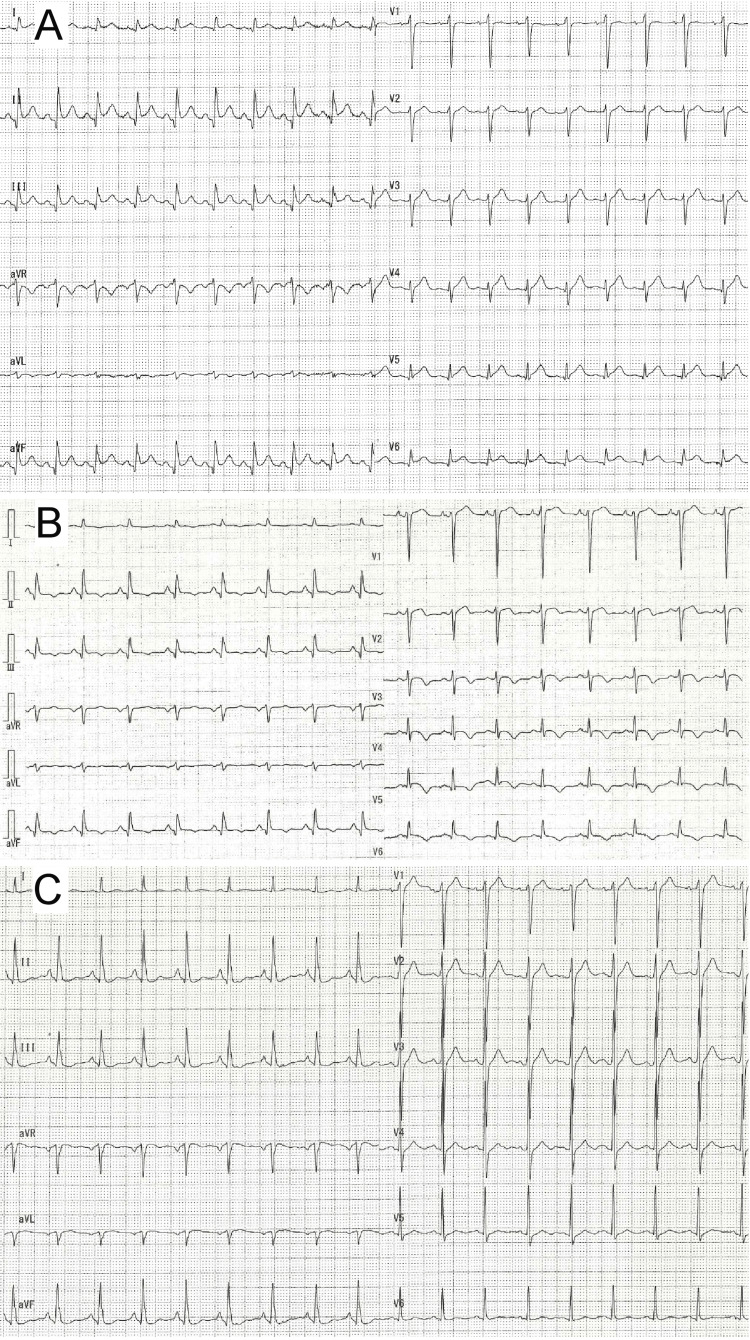
Electrocardiogram. (A) Electrocardiogram on arrival showing ST-segment elevation in leads II, III, aVF, and V6, and abnormal Q-waves in leads II, III, and aVF.  (B)  Electrocardiogram after two days showing T-wave inversion in leads II, III, aVF, V3, V4, V5, and V6. (C)  Electrocardiogram after five months showing improvement with non-specific ST-segment and T-wave changes.

Simultaneously, an electrocardiogram (ECG) revealed ST-segment elevation in leads II, III, aVF, and V6, and abnormal Q-waves in leads II, III, and aVF (Figure 2), suggestive of the existence of a concomitant ACS. His laboratory workup revealed a creatine kinase (CK) level of 316 IU/L (reference, <248 IU/L), a troponin I level of 7,556.3 pg/mL (reference, <26.2 pg/mL), and a brain natriuretic peptide level of 28.2 pg/mL (reference, <18.4 pg/mL). Transthoracic echocardiography showed a hypokinesis of the apical area. Left ventricular end-diastolic diameter/left ventricular end-systolic diameter was 50/27, and the estimated ejection fraction (EF) was 50%.

**Figure 3:**
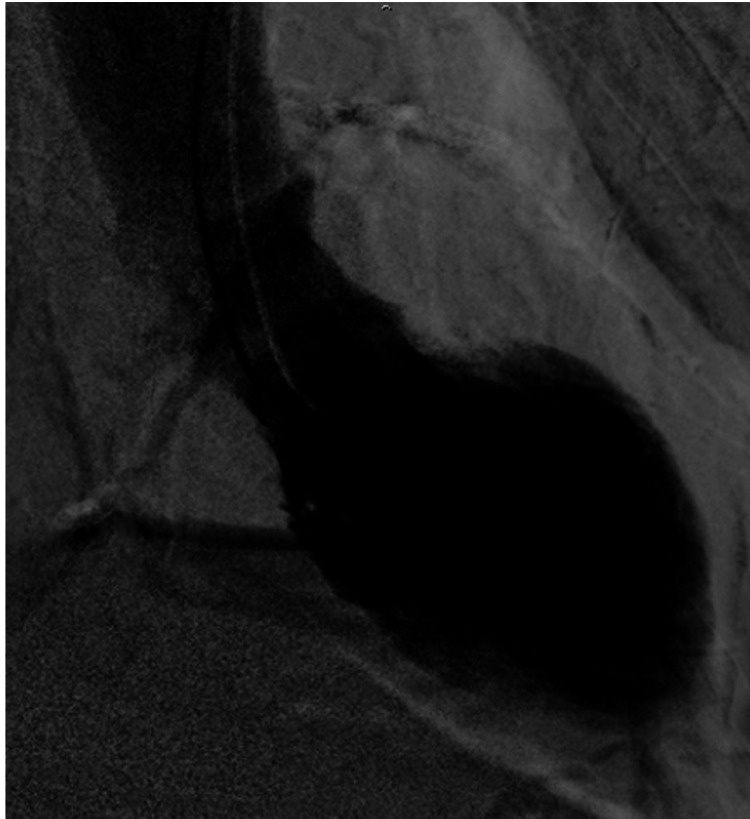
Left ventriculography. Apical akinesia and basal hyperkinesia.

Finally, coronary angiography showed no evidence of fresh obstructive coronary artery disease but left ventriculography showed apical akinesia and basal hyperkinesia, consistent with TC (Figure 3). He was treated with supportive care and his follow-up ECG (Figure 2C) and chest X-ray showed improvement (Figure 1C).

## Discussion

The patient’s chest pain was initially assessed to be secondary to recurrent pneumothorax. However, on further evaluation, ECG showed ST-segment elevation along with elevated troponin levels. Ultimately he was diagnosed with TC. The diagnosis of TC triggered by pneumothorax is challenging because pneumothorax and TC are rarely co-existent. To our knowledge, only six cases of pneumothorax and concomitant TC have been reported so far.

TC, also known as apical ballooning syndrome, stress cardiomyopathy, stress-induced cardiomyopathy, and broken-heart syndrome, is cardiomyopathy characterized by left ventricular systolic dysfunction in association with stressful conditions [2-4]. It is acute and usually reversible heart failure syndrome. Its presentation, including symptoms and ECG findings, mimics ACS, but there are no obstructive coronary artery lesions. It is important to discriminate TC from ACS because the treatment strategies of TC and ACS are completely different. TC accounts for 1-2% of patients who present with positive troponins and concern for ACS or ST-elevation myocardial infarction [4,5]. The possible mechanism includes catecholamine excess, which induces coronary artery spasm, microvascular dysfunction, and myocardial toxicity [6,7].

The diagnosis of TC triggered by pneumothorax is challenging because pneumothorax and TC are rarely co-existent, and pneumothorax itself can induce non-specific ECG changes, including ST-segment elevation [8]. Seven cases of pneumothorax and TC have been reported so far including our case (Table 1) [9-14]. Except for our case, all cases were female. Our case was mild with conservative treatment in both TC and pneumothorax. Three cases had tension pneumothorax, but their prognosis was favorable.

**Table 1:** Reported cases of Takotsubo cardiomyopathy and pneumothorax.

Age (years)	Gender	Symptoms	Pneumothorax	Electrocardiogram	Echocardiography	Authors
83	Female	Chest pain, progressive dyspnea, cough	Left-sided	Sinus tachycardia, ST elevation in V2–V5	Apical akinesis, basal hyperkinesis	Akashi et al. [9]
64	Female	Chest pain	Right-sided	Sinus tachycardia, ST elevation, T inversion in anterior and inferior leads	Global hypokinesis with apical ballooning, basilar hyperkinesis, ejection fraction 15%	Kumar et al. [10]
78	Female	Severe dyspnea	Left-sided, tension	Sinus tachycardia, ST elevation, T-wave changes in lateral leads	Global hypokinesis sparing the basal segments, apical ballooning, ejection fraction 13%	Gale et al. [11]
58	Female	Chest discomfort, cough, fever, progressive dyspnea	Right-sided, tension	ST elevation	Anterior wall hypokinesis with decreased ejection fraction	Mittal et al. [12]
58	Female	Progressive dyspnea, cough	Right-sided	Sinus tachycardia, ST elevation in V3–V5	Global akinesis sparing the base, ejection fraction 39%, small anterior pericardial effusion without an underlying tamponade physiology	Ghanimeh et al. [13]
76	Female	Chest pain, severe dyspnea, diaphoresis	Right-sided, tension	ST elevation in leads V2–V3	Not available at initial presentation	Chen et al. [14]
80	Male	chest pain, dyspnea	Left-sided	ST elevation in leads II, III, aVF, and V6, abnormal Q-waves in leads II, III, and aVF	Hypokinesis of the apical area, ejection fraction 50%	This case

TC and pneumothorax can be just a coincidence; however, a possible explanation for the association between these conditions is that stress and hypoxemia might induce catecholamine release, leading to TC. Besides TC, a case with concomitant spontaneous tension pneumothorax and acute myocardial infarction has been reported [15]. The authors alerted that multiple life-threatening diseases that present with similar symptoms can coexist, and a re-evaluation after performing the initial treatment for one of these diseases is important. Therefore, ECG and subsequent echocardiography should be considered to detect cardiac diseases, such as ACS or TC, when dealing with pneumothorax, because stress and hypoxemia might induce cardiac disease.

## Conclusions

ECG, if indicated by echocardiography, should be considered to detect concomitant heart disease when dealing with pneumothorax. TC should be recognized as a cardiac disease that can be caused by pneumothorax.

## References

[1] Hardy M, Cho A, Stavig A, Bratcher M, Dillard J, Greenblatt L, Schulman K (2018). Understanding frequent emergency department use among primary care patients. Popul Health Manag.

[2] Dote K, Sato H, Tateishi H, Uchida T, Ishihara M (1991). [Myocardial stunning due to simultaneous multivessel coronary spasms: a review of 5 cases]. J Cardiol.

[3] Tsuchihashi K, Ueshima K, Uchida T (2001). Transient left ventricular apical ballooning without coronary artery stenosis: a novel heart syndrome mimicking acute myocardial infarction. Angina Pectoris-Myocardial Infarction Investigations in Japan. J Am Coll Cardiol.

[4] Gianni M, Dentali F, Grandi AM, Sumner G, Hiralal R, Lonn E (2006). Apical ballooning syndrome or takotsubo cardiomyopathy: a systematic review. Eur Heart J.

[5] Kurowski V, Kaiser A, von Hof K (2007). Apical and midventricular transient left ventricular dysfunction syndrome (tako-tsubo cardiomyopathy): frequency, mechanisms, and prognosis. Chest.

[6] Paur H, Wright PT, Sikkel MB (2012). High levels of circulating epinephrine trigger apical cardiodepression in a β2-adrenergic receptor/Gi-dependent manner: a new model of Takotsubo cardiomyopathy. Circulation.

[7] Kassim TA, Clarke DD, Mai VQ, Clyde PW, Mohamed Shakir KM (2008). Catecholamine-induced cardiomyopathy. Endocr Pract.

[8] Krenke R, Nasilowski J, Przybylowski T, Chazan R (2008). Electrocardiographic changes in patients with spontaneous pneumothorax. J Physiol Pharmacol.

[9] Akashi YJ, Sakakibara M, Miyake F (2002). Reversible left ventricular dysfunction "takotsubo" cardiomyopathy associated with pneumothorax. Heart.

[10] Kumar A, Padala S, Morales DC, Swales H (2013). Broken lung and broken heart: a case of right pneumothorax resulting in Takotsubo cardiomyopathy. Conn Med.

[11] Gale M, Loarte P, Mirrer B, Mallet T, Salciccioli L, Petrie A, Cohen R (2015). Takotsubo cardiomyopathy in the setting of tension pneumothorax. Case Rep Crit Care.

[12] Mittal S, Garg P, Paliwal HP, Agarwal D, Saxena GN (2015). An unusual presentation of takotsubo cardiomyopathy in a setting of tension pneumothorax. IOSR-JDMS.

[13] Abu Ghanimeh M, Bhardwaj B, Aly A, Baweja P (2017). Takotsubo cardiomyopathy secondary to spontaneous right-sided pneumothorax. BMJ Case Rep.

[14] Chen WS, Hung MJ (2019). Tension pneumothorax-induced Takotsubo syndrome: a case report. Medicine (Baltimore).

[15] Arao K, Mase T, Nakai M, Sekiguchi H, Abe Y, Kuroudu N, Oobayashi O (2019). Concomitant spontaneous tension pneumothorax and acute myocardial infarction. Intern Med.

